# SARS-CoV-2 decreases malaria severity in co-infected rodent models

**DOI:** 10.3389/fcimb.2023.1307553

**Published:** 2023-12-13

**Authors:** Ana Fraga, Andreia F. Mósca, Diana Moita, J. Pedro Simas, Helena Nunes-Cabaço, Miguel Prudêncio

**Affiliations:** ^1^ Instituto de Medicina Molecular João Lobo Antunes, Faculdade de Medicina da Universidade de Lisboa, Lisboa, Portugal; ^2^ Católica Biomedical Research, Católica Medical School, Universidade Católica Portuguesa, Lisboa, Portugal

**Keywords:** co-infection, COVID-19, malaria, SARS-CoV-2, *Plasmodium*

## Abstract

Coronavirus disease 2019 (COVID-19) and malaria, caused by severe acute respiratory syndrome coronavirus 2 (SARS-CoV-2) and *Plasmodium* parasites, respectively, share geographical distribution in regions where the latter disease is endemic, leading to the emergence of co-infections between the two pathogens. Thus far, epidemiologic studies and case reports have yielded insufficient data on the reciprocal impact of the two pathogens on either infection and related diseases. We established novel co-infection models to address this issue experimentally, employing either human angiotensin-converting enzyme 2 (hACE2)-expressing or wild-type mice, in combination with human- or mouse-infective variants of SARS-CoV-2, and the *P. berghei* rodent malaria parasite. We now show that a primary infection by a viral variant that causes a severe disease phenotype partially impairs a subsequent liver infection by the malaria parasite. Additionally, exposure to an attenuated viral variant modulates subsequent immune responses and provides protection from severe malaria-associated outcomes when a blood stage *P. berghei* infection was established. Our findings unveil a hitherto unknown host-mediated virus-parasite interaction that could have relevant implications for disease management and control in malaria-endemic regions. This work may contribute to the development of other models of concomitant infection between *Plasmodium* and respiratory viruses, expediting further research on co-infections that lead to complex disease presentations.

## Introduction

The simultaneous presence of multiple pathogens in a host cell or organism is increasingly recognized as a critical aspect of infectious diseases. The impact of such co-infections on disease outcomes can range from harmful to inconsequential, or even beneficial, depending on the nature and extent of interactions that are established with and within the host (Devi et al., 2021). Indeed, the concurrent presence of multiple microorganisms can not only alter the dynamics of each infection, but also interfere with symptomatology and susceptibility to treatment (McArdle et al., 2018; Shen et al., 2019). Naturally, regions grappling with a high prevalence of various infectious diseases, such as malaria-endemic countries (Liu et al., 2022), are particularly burdened by co-infections, which assume an important role in shaping local disease dynamics. Thus, global events, such as the coronavirus disease 2019 (COVID-19) pandemic, can lead to unpredictable health consequences in these already susceptible populations.

Malaria remains a highly lethal infectious disease in low-income countries, ranking among the top 10 causes of death in these regions (Organization WH, 2020). According to the World Health Organization (WHO) estimates, over 200 million cases of malaria, resulting in more than 600,000 deaths, occurred in 2021, 95% of which in sub-Saharan Africa (World Health O, 2022). This devastating disease is caused by intracellular parasites of the *Plasmodium* genus and is transmitted between intermediate hosts by female *Anopheles* mosquitoes, which deposit *Plasmodium* sporozoites into the mammalian host’s skin during a blood meal (Phillips et al., 2017). These motile parasite forms enter the blood circulation and migrate towards the liver, where they traverse several cells before establishing a productive infection inside hepatocytes (Prudêncio et al., 2006). During this obligatory but clinically silent stage of infection, the parasite differentiates into exoerythrocytic forms (EEFs), which undergo an extensive replicative phase that culminates in the release of thousands of *Plasmodium* merozoites into the bloodstream (Prudêncio et al., 2006; Phillips et al., 2017). During the ensuing blood stage of infection, the parasites cyclically invade and burst red blood cells (RBCs), giving rise to malaria symptoms and allowing parasite transmission upon ingestion of gametocyte-infected RBCs by the mosquito vector (Meibalan and Marti, 2017). The clinical presentations of malaria can range from nonspecific flu-like symptoms in the case of uncomplicated malaria, to severe disease manifestations, such as cerebral malaria (Miller et al., 2013).

COVID-19 is caused by the severe acute respiratory syndrome coronavirus 2 (SARS-CoV-2), a betacoronavirus transmitted chiefly by aerosols (Gorbalenya et al., 2020). SARS-CoV-2 is responsible for respiratory tract infections, during which the virus enters its target cells through specific interactions between its Spike protein and host cell factors, most notably the angiotensin-converting enzyme 2 (ACE2) receptor (V’kovski et al., 2021). Generally, COVID-19 patients can experience a broad spectrum of symptoms. While some remain asymptomatic, others experience mild to moderate illness with general flu-like symptoms. However, severe cases involve an exacerbated and systemic immune response, with potential multiorgan failure and death (Wu et al., 2021; Merad et al., 2022). Inevitably, the concomitant presence of SARS-CoV-2 and *Plasmodium* in the same geographical area resulted in several instances of co-infection between these two pathogens. However, reported cases of such co-infections exhibited variable clinical presentations, ranging from expedited recoveries to worsened health outcomes (Kishore et al., 2020; Indari et al., 2021; Pusparani et al., 2021; Shahid et al., 2021; Wilairatana et al., 2021; Hussein et al., 2022; Ayisi-Boateng et al., 2023; Rathi et al., 2023).

Mammalian infection is a highly dynamic process involving an intricate array of host-pathogen interactions, often influenced by pathophysiologic conditions (reviewed in (Casadevall and Pirofski, 2018)). Since viral infection typically triggers a set of innate antimicrobial inflammatory cascades (Boechat et al., 2021; Yang et al., 2021), which, in turn, may impact responses against the different stages of *Plasmodium* infection (Jahiel et al., 1968; Jahiel et al., 1968a; Jahiel et al., 1970; Schofield et al., 1987; Mellouk et al., 1991; Liehl et al., 2014; Sanches-Vaz et al., 2019), we hypothesized that an initial exposure to SARS-CoV-2 infection might alter the host’s susceptibility to a subsequent infection by *Plasmodium* parasites. To test this hypothesis, two *in vivo* models of SARS-CoV-2 and *Plasmodium* co-infection were established, allowing the first rigorous and adequately controlled experimental investigation of this concomitant infection. While no evidence of retroactive modulation of viral infection by the *Plasmodium* parasite was found, our data showed that a primary viral exposure partially attenuated the liver-stage of *Plasmodium* infection and conferred protection against severe malaria-associated outcomes in co-infected animals. We further demonstrated that viral infection significantly modulates the circulating leukocyte landscape, prompting a distinct immune response in co-infected animals, relative to mice solely infected with *Plasmodium.* Our findings thus unveiled a variety of previously unknown host-mediated virus-parasite interactions, with a clear impact on the host’s susceptibility to infection and disease.

## Results

### Exposure of mice to an ancestral variant of SARS-CoV-2 impacts hepatic infection by the malaria parasite

**Figure 1:**
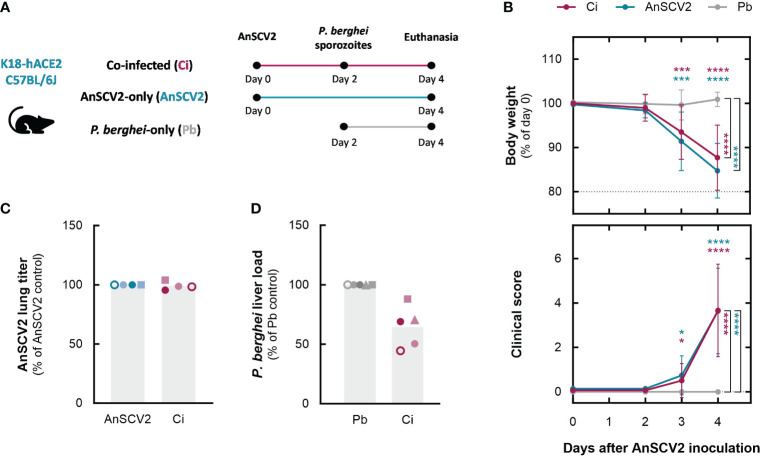
An ongoing AnSCV2 infection impacts a secondary liver infection by *P. berghei*. **(A)** Schematic representation of *Plasmodium* liver stage co-infection model. Depicted are the time points of infection with the ancestral SARS-CoV-2 virus strain (AnSCV2) and/or *P. berghei* sporozoites, and of euthanasia for organ collection. Experimental groups include mice inoculated with *P. berghei* sporozoites two days after exposure to AnSCV2 (Ci – pink), mice solely exposed to AnSCV2 infection (AnSCV2 – blue), and mice solely exposed to *P. berghei* sporozoite inoculation (Pb – grey). **(B)** Daily monitoring of body weight and signs of disease. Each symbol represents mean values of the group in every time point, and error bars represent the standard deviation of pooled data from four to five experiments (n=5 mice per group per experiment, N=4-5). Dotted horizontal lines represent the weight threshold for euthanasia (top graph). **(C)** AnSCV2 pulmonary infection quantified by virus titration and plaque-forming assay in Vero CCL-81 cells 4 days post virus inoculation. Each symbol represents mean values for the group in each experiment (n=3-5 mice per group per experiment) and bars represent the mean values for the pooled data (N=4). **(D)** *P. berghei* liver infection quantified by RT-qPCR 46 h after sporozoite injection. Each symbol represents mean values for the group in each experiment (n=5 mice per group per experiment) and bars represent the mean values for the pooled data (N=5). The statistical significance of differences between pairs of groups was assessed a two-way analysis of variance (ANOVA) followed by the Sidak’s test for multiple comparisons in **(B)**, and by unpaired t tests in **(C)** and **(D)** (* p<0.05, *** p<0.001, **** p<0.0001). Coloured asterisks indicate differences relative to *P. berghei-*single infected mice.

To assess the impact of a SARS-CoV-2 infection on the asymptomatic, yet obligatory, hepatic stage of *P. berghei* infection, ACE2-humanized mice were inoculated with 3x10^4^ *P. berghei* sporozoites (Sanches-Vaz et al., 2019). The malaria parasites were inoculated two days after the animals had been exposed to an ancestral variant of SARS-CoV-2 (AnSCV2) that causes a severe disease phenotype (**Figure 1A**), a time point at which viral replication in the lungs is at its peak (Winkler et al., 2020). Mice single-infected with either AnSCV2 or *P. berghei* sporozoites were included as controls. Progression of the viral disease was assessed by daily monitoring of body weight changes and signs of disease until euthanasia. Contrarily to the *P. berghei-*only infected mice, both groups of animals exposed to AnSCV2 displayed identical weight losses starting on day 3 after inoculation of the virus (**Figure 1B**). Likewise, quantification of infectious viral particles in the lungs revealed no significant differences in the viral titers of AnSCV2-single and co-infected mice (**Figures 1C** and **S1A**). Together, these findings indicate that the subsequent liver infection by *P. berghei* did not have a discernible effect on AnSCV2-associated disease progression or viral replication in the lungs. Conversely, to evaluate the impact of a primary exposure to AnSCV2 on the host’s susceptibility to the initial stage of *P. berghei* infection, mouse livers were collected 46 h post sporozoite inoculation and parasite load was quantified by reverse transcription quantitative PCR (RT-qPCR). Although some variability is observed, our results show that the *P. berghei* liver load of animals previously exposed to AnSCV2 tended to be lower than that of mice solely infected with *P. berghei*, with 1 in 5 independent experiments reaching statistical significance (**Figures 1D** and **S1B**).

**Figure 2:**
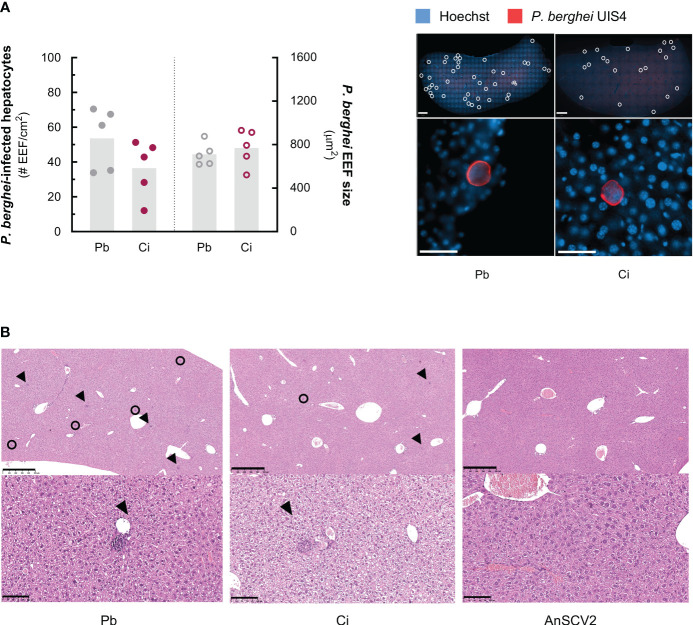
Co-infection decreases the number of *P. berghei*-infected hepatocytes in an infiltration independent way. **(A)** Left: number of *P. berghei*-infected hepatocytes per cm^2^ of liver (left Y-axis) and size of exoerythrocytic forms (EEF) in µm^2^ (right Y-axis) of liver slices collected from *P. berghei-*single infected mice (Pb – grey symbols) and co-infected mice exposed to AnSCV2 two days earlier (Ci – pink symbols), 46 h post sporozoite inoculation, quantified by immunofluorescence microscopy analysis. Each dot represents the mean value per mouse, bars represent the mean values for the group from one experiment (n=5 mice per group, N=1). Right: representative immunofluorescence microscopy images of whole liver slices (top panels; 1x magnification mosaic; scale bar, 1 mm) and EEFs (bottom panels; 40x magnification; scale bar, 50 μm) from each experimental group. Individual *P. berghei* EEFs are highlighted (white circles; upper panels). Blue: Hoechst (nuclei); red: *P. berghei* upregulated in infective sporozoites gene 4 (UIS4; parasitophorous vacuole membrane). **(B)** Representative histology images of haematoxylin and eosin-stained liver sections from *P. berghei-*single, co- and AnSCV2-single infected mice from one experiment (N=1). *P. berghei* EEFs (black circles) and inflammatory cell infiltrates (black arrows) are highlighted (upper panels; 5x magnification; scale bar, 500 μm). Microgranulomas (black arrows) mainly composed of mononuclear inflammatory cells are observed in *P. berghei*-only and co-infection conditions, but not in AnSCV2-only infected mice (lower panels; 20x magnification; scale bar, 100 μm). The statistical significance of differences between groups in **(A)** was assessed employing an unpaired t test.

The reduced *P. berghei* liver load observed in animals previously exposed to AnSCV2 might result from a reduction in the number of parasites that successfully infect hepatocytes or from an inhibition of parasite growth inside the hepatic cells. To assess this, liver sections from single- and co-infected mice from a representative experiment were collected 46 h after sporozoite inoculation and analysed by immunofluorescence microscopy. Our results showed that co-infected mice tended to exhibit a lower number of *P. berghei*-infected hepatocytes than the single infected controls, whereas the size of the developing EEFs was similar for both groups (**Figure 2A**). Collectively, these results suggest that the AnSCV2-associated reduction in *P. berghei* hepatic infection stemmed primarily from a decrease in the number of *P. berghei*-infected hepatocytes, rather than from an impairment of the parasite’s intrahepatocytic development. To understand whether this decrease could be attributed to a generalized, AnSCV2-induced, liver lesion, histopathology analysis of liver sections collected from a representative experiment was conducted. Our results revealed the absence of significant deviations from normal hepatic architecture for any of the experimental animals, with only the two *P. berghei-*infected groups exhibiting rare focal inflammatory infiltrates (**Figure 2B**). These observations indicate that the observed reduction in parasite-infected hepatocytes in co-infected mice could not be attributed to an exacerbated immune cell infiltration or liver damage triggered by exposure to AnSCV2.

### Asymptomatic viral infection protects mice from severe malaria-associated outcomes

While the initial hepatic stage of infection constitutes a major asymptomatic bottleneck in the *Plasmodium* life cycle (Graumans et al., 2020), the parasite’s progression into the blood stage can lead to the development of disease symptoms, which may include severe syndromes such as cerebral malaria (White, 2014).

The combination of C57BL/6J mice and *P. berghei* ANKA parasites is the most commonly employed model of experimental cerebral malaria (ECM), a neurological syndrome characterized by microvascular pathology and sequestration of parasitized red blood cells in the mouse brain (Zuzarte-Luis et al., 2014a). However, K18-hACE2 C57CL/6J mice do not constitute an adequate model for the investigation of this phenotype in the context of a co-infection with SARS-CoV-2, as the overexpression of hACE2 in the brains of these mice has been linked to lethal viral neurodissemination and encephalopathy (Fumagalli et al., 2022), which is not typically observed during human SARS-CoV-2 infection. To overcome this constraint, a more physiologically relevant model was established, employing a mouse-adapted SARS-CoV-2 variant (maSCV2), which is fully capable of infecting wild-type mice (Leist et al., 2020). Furthermore, the milder, non-lethal nature of this viral infection (Leist et al., 2020) makes it more representative of the current course of the COVID-19 epidemiological situation, in which a large proportion of patients exhibit asymptomatic or mild disease.

The attenuated nature of maSCV2 infection and its effect on the liver stage of the *Plasmodium* life cycle was initially assessed in a 4-day experimental setting identical to that employed in our previous experiments, in which wild-type C57BL/6J mice were inoculated with 3x10^4^ *P. berghei* sporozoites two days after viral infection (**Figure S2A**). Monitoring of disease signs confirmed the absence of severe disease in maSCV2-infected mice throughout the experimental time frame, and RT-qPCR analysis of liver samples revealed a tendency for a lower hepatic parasite load in co-infected animals, compared to the *P. berghei-*single infected mice (**Figures S2B, S2C**).

**Figure 3:**
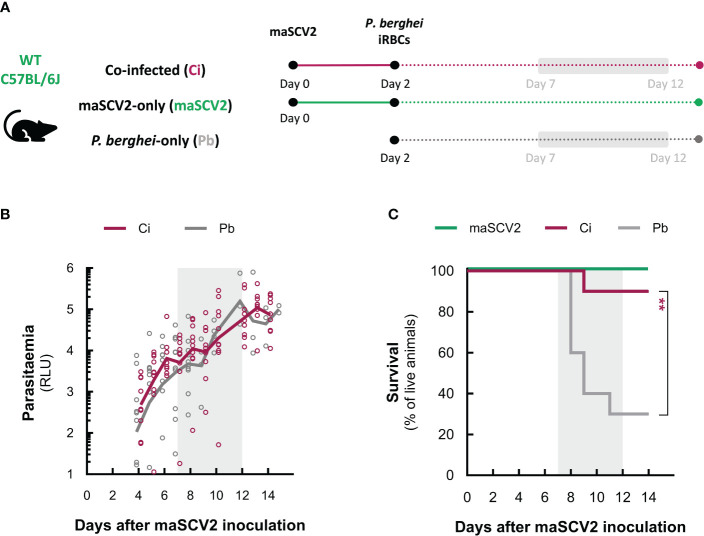
An attenuated SARS-CoV-2 infection protects against severe malaria pathology. **(A)** Schematic representation of the attenuated co-infection model. Depicted are the time points of infection with the mouse-adapted SARS-CoV-2 strain (maSCV2) and/or *P. berghei*-infected red blood cells (iRBCs), and the period during which parasitaemia and survival were monitored. Experimental groups include mice inoculated with *P. berghei*-iRBCs two days after exposure to maSCV2 (Ci – pink), mice solely exposed to maSCV2 infection (maSCV2 – green), and mice inoculated solely with *P. berghei*-iRBCs (Pb – grey). **(B)** *P. berghei* parasitaemia from day 2 post-maSCV2 inoculation onwards. Each symbol represents one mouse and lines represent the mean values for the group from a pool of two independent experiments (n=5 mice per group per experiment, N=2). **(C)** Daily monitoring of mouse survival. Lines represent the percentage of live mice from a pool of two independent experiments in C (n=5 mice per group per experiment, N=2). The statistical significance of differences was assessed by unpaired t tests in **(B)** and the Mantel-Cox (log rank) test between Ci and Pb in **(C)** (** P<0.01). Grey-shaded areas in **(A-C)** indicate the 5-day window of ECM development.

In view of these results, we then sought to investigate potential effects of exposure to maSCV2 infection on the erythrocytic stage of *P. berghei* infection, independently of pre-erythrocytic events. To this end, the liver stage of the parasite’s life cycle was bypassed by inoculating 1x10^6^ *P. berghei*-infected red blood cells into naïve or maSCV2-infected mice, followed by daily monitoring of parasitaemia and survival (**Figure 3A**). Our results demonstrate that, although *P. berghei* parasitaemia in co-infected mice was largely indistinguishable from that of animals solely infected with *P. berghei* (**Figure 3B**), only 1 out of 10 co-infected animals, as opposed to 7 out of 10 P*. berghei-*single infected mice, exhibited signs of ECM (**Figure S3**), with the former surviving significantly longer than the latter in two independent experiments (**Figure 3C**). Collectively, these findings show that a primary exposure to an asymptomatic maSCV2 infection protected against severe malaria-associated symptomatology and death, and that this protection was independent of the parasite burden in the blood.

### Primary exposure to maSCV2 influences immune responses to *P. berghei* blood stage infection

**Figure 4:**
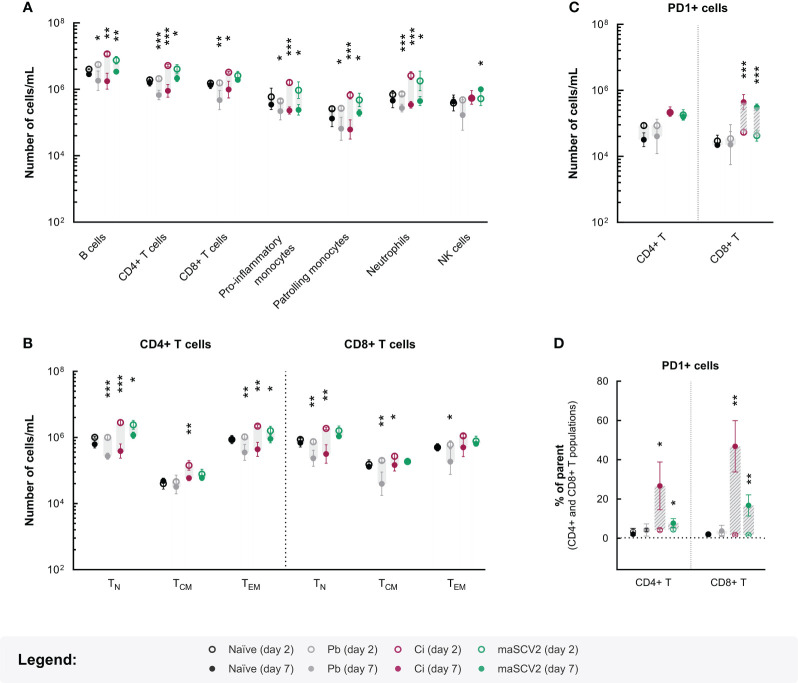
Impact of maSCV2 and/or *P. berghei* blood infection on circulating immune cells. Mean values for the numbers of B, CD4+ T, CD8+ T, monocyte, neutrophil and natural killer (NK) cells **(A)**, the naïve (T_N_), central memory (T_CM_) and effector/effector memory (T_EM_) CD4+ T and CD8+ T cells **(B)**, and the PD-1+ CD4+ T and CD8+ T cells **(C)** on day 2 (open circles) and day 7 (closed circles), in each condition. Scatter dot plots for the proportion of PD-1+ cells **(D)** within the CD4+ T and CD8+ T cell compartments on day 2 (open circles) and 7 (closed circles), in each experimental condition. The grey shaded area and the dashed grey shaded area highlight, respectively, decreases or increases in cell population numbers or frequencies between day 2 and 7. Experimental groups include naïve mice (black symbols), mice solely infected with *P. berghei*-infected red blood cells (Pb – grey symbols), mice exposed to maSCV2 infection 2 days prior to *P. berghei* inoculation (Ci – pink symbols) and mice exposed only to maSCV2 infection (maSCV2 – green symbols). Each symbol represents the mean values for the group and error bars represent the standard deviation from one experiment (n=4-5 mice per group). Statistical significance of differences in cell numbers or frequencies between day 2 and 7 for each experimental group was assessed by employing paired t tests (* p<0.05, ** p<0.01, *** p<0.001).

Since the observed increase in survival of co-infected mice relative to their *P. berghei*-single infected counterparts was not influenced by the parasite burden in the blood, we hypothesized that this protective effect might be associated with the immune events that lead to brain pathology (Grau and Wassmer, 2017; Ghazanfari et al., 2018). To investigate the immune response to blood-stage infection of co- and single-infected hosts, blood samples were collected immediately before inoculation of *P. berghei*-iRBCs and five days later, and the immune profile of circulating leukocytes was assessed by multi-parameter flow cytometry (**Figure S4**). Although some variability between experimental groups was observed, the comprehensive analysis of the main cell populations involved in innate and adaptative immunity clearly revealed that animals exposed to maSCV2 displayed increased numbers of most circulating cell types relative to non-infected mice, at the time of parasite inoculation (two days after viral infection; **Figures 4A** and **S5A**). However, by day 7, this early increase in leukocyte numbers appeared to be completely resolved in the maSCV2-single infected animals, while both groups of parasite-infected hosts displayed general decreases in the number of circulating immune cells relative to naïve mice, suggesting potential cell trafficking to tissue compartments in the latter (**Figures 4A** and **S5B**). Knowing that *P. berghei-*induced depletion of circulating leukocytes tends to aggravate as blood stage infection progresses (Nunes-Cabaço et al., 2022), these results not only emphasise the distinct fate of the circulating immune populations during the course of each single infection, but also inform about the predominance of parasite-directed responses during later stages of co-infection. Nevertheless, despite the considerable similarities between the circulating immune landscape of co-infected and *P. berghei*-single infected mice on day 7, the former seem to exhibit an intermediate phenotype in circulating CD8+ T and NK cell populations when compared to both groups of single-infected control mice (**Figures 4A** and **S5B**). These results suggest that the initial encounter of the co-infected animals with maSCV2 modified the subsequent patterns of response to parasitic infection relative to that observed in animals exposed solely to *P. berghei* infection.

To gain further insight into the responses occurring within the CD4+ helper T and CD8+ cytotoxic T cell compartments, the expression of activation and memory-associated markers was assessed. In accordance to our previous findings (**Figures 4A** and **S5B**), on day 7, the co-infected animals exhibited cell dynamics within the CD4+ T cell compartment comparable to those of mice infected solely with *P. berghei* (**Figures 4B**, **S5D** and **S6B**). Conversely, a noticeable disparity was observed between the co- and *P. berghei*-single infected animals regarding activated CD8+ T cell subpopulations. Specifically, the significant declines in numbers of circulating central memory (T_CM_) and effector/effector memory (T_EM_) CD8+ T cells seen in the hosts infected only with *P. berghei* were not evident in co-infected animals, suggesting increased production and/or proliferation, or conversely, reduced mobilization of these activated CD8+ T cells in the latter group (**Figures 4B** and **S5D**). Moreover, the increased proportion of CD8+ T_EM_ cells in the circulation of co-infected animals, in comparison to both single-infected control mice (**Figure S6B**), underscores the impact of primary viral exposure on the dynamics of CD8+ T cell responses when the host is subsequently exposed to parasitic infection.

In light of these results and recognizing the crucial role of programmed cell death-1 (PD-1), a co-stimulatory molecule found in activated T cells, in restraining unbridled immune responses that could lead to tissue damage (Sharpe and Pauken, 2018), the expression of this maker in CD4+ and CD8+ T cells was analysed. Our results show that PD-1-expressing (PD-1+) T cells emerged in the bloodstream of both groups of maSCV2-infected animals between day 2 and 7, displaying a noticeable increase within the CD8+ T cell population at day 7, while remaining mostly unaltered in the *P. berghei*-single infected hosts throughout the same time course (**Figures 4C**, **S5E** and **S5F**). Remarkably, these PD-1+ cells were highly enriched in both the CD4+ and, to an even greater extent, the CD8+ T cell compartment of co-infected animals, compared to either group of single-infected mice (**Figures 4D**, **S6C** and **S6D**). Taken together, these findings suggest that PD-1 might be significantly upregulated on T cells during co-infection, compared to single infection with *P. berghei* (**Figures 4B** and **S5D**). Ultimately, these altered immune cell dynamics in co-infected mice might translate into hampered immune-mediated brain pathology and grant the observed protection from severe malaria-associated outcomes.

## Discussion

Concomitant infection by multiple pathogens is not an uncommon occurrence, particularly in areas affected by a high burden of various infectious diseases, such as malaria-endemic countries (Liu et al., 2022). Besides posing major diagnostic and therapeutic challenges, co-infection can lead to complex pathogen-pathogen and host-pathogen interactions, possibly altering overall susceptibility to infection and disease (McArdle et al., 2018). In light of these complexities, gaining a deeper understanding of how pathogens interact with each other and with the host, and of how they influence the outcomes of disease, becomes crucial. Since the start of the COVID-19 pandemic, several observational studies attempted to describe the potential consequences of the co-endemicity between *Plasmodium* and SARS-CoV-2 (Wilairatana et al., 2021; Hussein et al., 2022). However, the associations established in these studies are of difficult interpretation and markedly influenced by sociodemographic factors. As such, the consequences of this co-infection on the host’s susceptibility to each microorganism and on pathogenesis remain largely unknown. Knowing that SARS-CoV-2 infection elicits a marked immune response, we postulated that a primary infection with this virus could influence *Plasmodium*’s ability of successfully establish infection and/or influence its natural progression in the mammalian host.

Like in most viral infections, SARS-CoV-2 replication in host cells is associated with the activation of intracellular signalling cascades that culminate in the production of innate interferons (IFNs) (Boechat et al., 2021). During the first stages of response against respiratory infections, these IFNs, consisting mainly of type I and type III IFNs, are responsible for the establishment of a cellular state of microbial resistance (cell autonomous immunity) and the production of various inflammatory mediators. These mediators are released both locally and systemically, allowing the activation and recruitment of subsequent immune responses with the goal of limiting the spread of the infection (MacMicking, 2012; Boechat et al., 2021). Severe disease-causing variants of SARS-CoV-2, such as AnSCV2, often induce an exacerbated activation of these inflammatory cascades, leading to a phenomenon known as “cytokine storm”, responsible for systemic damage of multiple tissues and organs (Boechat et al., 2021; Yang et al., 2021). Although liver pathology has been reported in animal models of SARS-CoV-2 infection (Yinda et al., 2021; Shou et al., 2021), our histopathology analysis of livers from AnSCV2-infected mice did not reveal remarkable signs of inflammatory hepatic lesions, suggesting that the observed reduction in *Plasmodium* liver infection of co-infected animals relative to *P. berghei*-single infected controls could not be attributed to AnSCV2-mediated liver damage. Nonetheless, exogenous administration or induction of type I IFNs has been reported to reduce hepatic infection by *Plasmodium* parasites (Jahiel et al., 1968; Jahiel et al., 1968a; Jahiel et al., 1970; Liehl et al., 2014). Likewise, IFN-γ, a second line IFN produced by activated lymphocytes, has been associated with an impairment of *Plasmodium* infection *in vitro* (Mellouk et al., 1991) and *in vivo* (Schofield et al., 1987; Sanches-Vaz et al., 2019). Since these inflammatory cascades are largely recapitulated in the ACE2-humanized murine model employed in our study (Winkler et al., 2020), it can be hypothesized that the production of these innate and adaptative inflammatory mediators during primary exposure to AnSCV2, and their subsequent upregulation in the circulation of co-infected mice, might be implicated in the observed reduction in the number of hepatic parasites. However, the exact mechanism by which primary exposure to AnSCV2 modulates hepatic infection by *P. berghei* requires further investigation.

Currently, severe forms of SARS-CoV-2 primarily affect specific risk groups, such as older, chronically ill or immunocompromised individuals, while overall occurrences in the remaining groups have decreased (Semenzato et al., 2022; Zsichla and Müller, 2023). In view of this epidemiological progression, a more attenuated and, thus, more clinically relevant co-infection model was established, employing an attenuated maSCV2. Although exposure to maSCV2 had no statistically significant effect on liver infection by *P. berghei*, its impact on malaria pathogenesis was clear when this stage was bypassed and the blood stage of infection was assessed. Our results showed that exposure to maSCV2 protected co-infected mice from severe neurological symptoms and significantly enhanced their overall survival, relative to *P. berghei*-single infected mice. Interestingly, these effects occurred independently of any alteration in parasite replication in the blood of co-infected animals, but were accompanied by significant modifications in the dynamics of NK and CD8+ T cells, two major immune cell populations involved in general responses against intracellular pathogens (Thakur et al., 2019) and implicated in ECM pathophysiology (Grau and Wassmer, 2017; Ghazanfari et al., 2018), which were not observed in mice solely infected with *P. berghei*.

Numerous studies have demonstrated that concurrent infections modulate malaria-associated outcomes (Gay et al., 2002; Davenport et al., 2016; Sanches-Vaz et al., 2019; Shen et al., 2019; Mahittikorn et al., 2021). For instance, the lactate dehydrogenase-elevating virus (LDV), a macrophage-infecting virus which induces a persistent asymptomatic infection and prolonged viraemia in mice, has been reported to confer protection against *P. berghei* ANKA-induced ECM (Hassan et al., 2020). This protection was attributed to the robust LDV-induced type I IFN response, leading to a functional impairment of key immune cell populations involved in ECM neuropathology. On the other hand, concomitant infection of *P. berghei* with the mosquito-borne Chikungunya virus (CHIKV) also conferred protection against ECM through a IFN-γ-mediated retention of pathogenic CD8+ T cells in the spleen, preventing their migration to the brain (Teo et al., 2018). However, unlike SARS-CoV-2, infection by CHIKV spreads via the bloodstream, infecting various organs, including lymphoid tissues, where it can establish persistent infection (Labadie et al., 2010; Hong et al., 2019). Besides the direct effect of soluble mediators on cell responses implicated in ECM development, this syndrome has been shown to be highly dependent on a synchronous sequestration of a critical number of infected RBCs and activated cytotoxic CD8+ T cells within the brain microcirculation, along with the establishment of a local inflammatory environment (McQuillan et al., 2011; Sato et al., 2019). Therefore, a decoupling of these critical events could result in the failure to trigger the inflammatory responses associated with brain pathology. Our blood immunophenotyping data showed that challenge with *P. berghei* blood stage parasites led to distinct immune responses in mice primarily exposed to maSCV2 compared to the *P. berghei-*single infected hosts. Specifically, not only did the former exhibit a less pronounced decrease in circulating NK and CD8+ T cells than the latter, but they also did not show the decline in activated central memory (T_CM_) and effector/effector memory (T_EM_) CD8+ T cells observed in the *P. berghei*-single infected animals. Although a heightened production or proliferation of these cell types during co-infection might account for these findings, it is also plausible that cell mobilization to inflamed tissues, such as the brain, is reduced in co-infected animals, thus disrupting the inflammatory events that lead to ECM. Furthermore, our results suggest that co-infection led to an upregulation of programmed cell death-1 (PD-1) in circulating T cells. Since PD-1 expression by T cells has been associated with reduced pro-inflammatory responses and increased protection against ECM (Hafalla et al., 2012; Wang et al., 2018), it can also be hypothesized that initial exposure to the maSCV2 could have increased survival by functionally impairing these cells and decreasing immune-mediated pathology. Nonetheless, further investigation is required to fully understand the underlying mechanisms of the observed protection against ECM in the context of a localized respiratory viral infection, as well as the functional role of each immune cell type in this phenotype.

To the best of our knowledge, this is the first report to experimentally address the impact of primary exposure to SARS-CoV-2 on the outcomes of a subsequent *Plasmodium* infection in relevant *in vivo* models. Our results suggest that early inflammatory events triggered by SARS-CoV-2 might lead to elimination of hepatic *Plasmodium* parasites when an exacerbated antimicrobial response is established. Besides, when a controlled and effective anti-viral immune response occurs, the differential activation of circulating immune cells might interfere with the establishment of the brain-localized inflammatory events required for the development of ECM. Nonetheless, the specific host factors involved in the observed phenotypes remain unknown and should be further investigated.

Given the current global epidemiologic status of malaria, co-infections between *Plasmodium* and other pathogens, including respiratory viruses, are bound to occur. As such, our work paves the way for the development of additional co-infection models between *Plasmodium* and viruses responsible for respiratory diseases, thus allowing the elucidation of interactions arising between two major groups of pathogens, in a rigorous and controlled manner, ultimately filling an important knowledge gap regarding complex disease presentations.

## Materials and methods

### Experimental animals

Wild-type C57BL/6J mice purchased from Charles River Laboratories were housed in the specific-pathogen-free (SPF) facilities of Instituto de Medicina Molecular João Lobo Antunes (IMM JLA). Heterozygous K18-hACE2 C57BL/6J mice acquired from The Jackson Laboratory were bred and housed in the same facility. Experimental animals (8-12 weeks old) were handled in biosafety level 3 (BSL-3) conditions. All experimental work involving animals was performed in accordance with the current legislation (Directive 2010/63/Eu and Decreto-lei 113/2013) and followed the Federation of European Laboratory Animal Science Associations (FELASA) guidelines. All procedures are included in the research project “Transgenic hACE2 mice model of SARS-CoV-2 infection”, licensed by Direção Geral de Alimentação e Veterinária (license number: 001878-2021).

### Pathogens and infections

The European ancestral strain of SARS-CoV-2 virus (herein referred to as AnSCV2) was isolated at iMM JLA from a clinical sample (European Nucleotide Archive sample accession SAMEA6844881). A mouse-adapted SARS-CoV-2 virus strain (herein referred as maSCV2) obtained from the BEI Resources repository (NR-55329) was generously provided by Dr. Gonçalo Bernardes (IMM JLA). All viral stocks were propagated using Vero cells from ATCC^®^ (CCL-81™) and stored at -80°C. Aliquots of AnSCV2 or maSCV2 viral stocks were thawed and experimental mice were inoculated with 1-2.5x10^4^ plaque-forming units (PFU) by intranasal (i.n.) instillation, under light isoflurane anaesthesia, two days prior to *Plasmodium* infection. Transgenic luciferase-expressing *P. berghei* ANKA rodent malaria parasites (Franke-Fayard et al., 2005) (hereafter referred to as *P. berghei*) were kindly provided by Chris Jansen (Leiden University Medical Centre). *P. berghei* sporozoites were obtained from the salivary glands of infected *Anopheles stephensi* mosquitoes reared and infected at IMM-JLA. Mice were infected by retro-orbital intravenous (i.v.) injection of 3x10^4^ sporozoites, under light isoflurane anaesthesia. *P. berghei*-infected red blood cells (iRBCs) were obtained from a donor mouse previously infected with 1x10^6^ iRBCs from a frozen stock and experimental mice were infected by intraperitoneal (i.p.) injection of 1x10^6^ iRBCs. All experiments with SARS-CoV-2 viruses were performed in IMM-JLA certified BSL3 laboratories, following international safety guidelines.

### Assessment of disease progression

Body weight and clinical signs of disease were monitored daily following the viral infection, until euthanasia. Virus-related signs of disease were recorded using a modified version of the clinical score described in (Moreau et al., 2020). Briefly, a score ranging from 0 to 7 points was employed based on the evaluation of signs of disease, including back arching (0-1 points), ruffled fur (0-2 points), activity (0-3 points) and respiratory difficulties (0-1 points). Experimental cerebral malaria (ECM) was assessed using an adapted version of the Rapid Murine Coma and Behaviour Scale (Carroll et al., 2010), in which each of the seven signs observed (gait, balance, motor performance, body position, limb strength, touch escape and grooming) ranged from 0 to 2 points, up to a maximum of 14 points. In both scores, higher total values are associated with aggravated disease states. Euthanasia was performed whenever a 20% decrease in body weight was reached and/or severe signs of disease (total score higher than 5 out of 7 or 10 out of 14, respectively) were recorded.

### Assessment of SARS-CoV-2 pulmonary infection

Mice were euthanized by isoflurane overdose and the lungs were harvested 4 days post viral inoculation. Each left lung was mechanically homogenized in 3 mL of homogenization medium (medium compositions in **Table S1**), clarified by centrifugation (300 g for 3 min at room temperature (RT)) and stored at -80 °C until further analysis.

Infectious AnSCV2 viral particles were titrated by a plaque-forming assay, using Vero CCL-81 cells. The adherent cells were seeded into 6-well plates at a density of 8x10^5^ cells/well in growth media and incubated for 24 h. Lung homogenates were serially diluted ten-fold in maintenance medium and inoculated into duplicate wells after growth medium removal. The plates were incubated in a CO_2_ incubator for 1 h and gently rocked every 15 min. The inoculum was then discarded, 2 mL of overlay medium were added, and the plates were incubated for 4 days at 37 °C. After removal of the overlay medium, the cells were fixed with 4% formaldehyde/PBS for 1 h and stained with 0.1% toluidine blue. Viral plaques were counted using a magnifying glass and the viral titer calculated as plaque forming units/mL, according to: PFU/mL = average number of plaques × $\frac{1}{\text{dilution}}$ × $\frac{1}{\text{inoculum }\left(\text{mL}\right)\;}$ . Total left lung viral titers were further normalized to the respective lung weight.

Infection by maSCV2 was confirmed by reverse transcription quantitative PCR (RT-qPCR). Viral RNA was extracted from homogenized samples using the NYZ Viral RNA Isolation kit (NYZTech), according to the manufacturer’s recommendations. Final RNA concentration was quantified using a NanoDrop DR 2000 Spectrophotometer (Thermo Fisher Scientific) and complementary DNA (cDNA) was synthesized from 1 μg of RNA using the NZYTech cDNA synthesis kit (NZYTech) according to the manufacturer’s recommendations, and employing the following thermocycling parameters: 25°C for 10 min, 55°C for 30 min, and 85°C for 5 min. cDNA was amplified by qPCR using an Applied Biosystems 7500 Fast Real-Time PCR System, in which each 10 µL reaction contained 5 µM of Power SYBR Green PCR Master Mix (Applied Biosystems) and 0,5 µM of each primer (forward and reverse), and employing the thermocycling parameters: 50 °C for 2 min, 95 °C for 10 min, 40 cycles at 95 °C for 15 s and 60 °C for 1 min with melting stage of 95 °C for 15 s, 60 °C for 1 min and 95 °C for 30 s. The mouse housekeeping gene hypoxanthine guanine phosphoribosyl transferase (*Hprt*) was used for normalization and the comparative CT (ΔΔCT) method was employed for all gene expression analyses. The primers employed in RT-qPCR are listed in **Table S2**.

### Assessment of *P. berghei* liver infection

Mice were euthanized by isoflurane overdose and the livers were collected 46 h after sporozoite inoculation (Wang et al., 2018). *P. berghei* liver load was assessed by RT-qPCR and further characterized by immunofluorescence microscopy analysis of liver slices.

For RT-qPCR analyses, the caudate and right liver lobes (0.7–0.9 mg of liver sample) were mechanically homogenized in either TRIzol reagent (BioRad) or denaturing solution (4 M guanidine thiocyanate; 25 mM sodium citrate pH 7; 0.5% N-lauroylsarcosine and 0.7% β-mercaptoethanol in DEPC-treated water) and left 1 h at RT to allow viral inactivation (Winkler et al., 2020), as a safety measure before retrieving the samples from the BSL-3 facility. RNA was isolated either by TRIzol RNA extraction, employing the manufacturer’s instructions, followed by ethanol purification (Green and Sambrook, 2012) or using the Qiagen RNA extraction kit. RNA quantification, cDNA synthesis and qPCR were conducted as described above. Parasite load was assessed using primers specific for *P. berghei* 18S rRNA (**Table S2**) and the mouse housekeeping gene *Hprt* was used for normalization by the comparative CT (ΔΔCT) method.

For immunofluorescence microscopy analysis, the left liver lobe was fixed in 4% paraformaldehyde (SantaCruz Biotechnology) overnight at 4-8 °C and sectioned into 50 μm thick slices using a vibrating blade microtome (VT1200S, Leica), followed by staining and analysis using an adapted version of (Mendes et al., 2017). Briefly, four liver sections with similar areas were selected from each animal at regular intervals throughout the lobe to ensure representative sampling (2.32 cm^2^ *vs.* 2.55 cm^2^ of average liver area analysed for *P. berghei*-single infected and co-infected animals, respectively), and were incubated in permeabilization/blocking solution containing 2% Albumin Bovine Serum (VWR Chemicals) and 0.5% Triton-X100 (Carl Roth) in 1x PBS for 1 h at RT. The parasite’s surrounding membrane was stained with a goat anti-upregulated in infective sporozoites gene 4 (UIS4) primary antibody (1:300 dilution; Siagen) overnight at 4-8°C, followed by incubation with donkey Alexa Fluor 488 anti-goat antibody (1:500 dilution; Invitrogen) and Hoechst 33342 double stranded DNA dye (1:1000 dilution; Invitrogen) for 2 h at RT. Primary and secondary antibodies, as well as the DNA dye, were diluted in permeabilization/blocking solution and each antibody incubation was washed thrice with 1x PBS with 5 min agitation. The liver slices were mounted on microscope slides with Fluoromount G (Invitrogen) and image acquisition was carried out using a Zeiss Cell Observer inverted widefield fluorescence microscope. Image processing for number and size quantification of exo-erythrocytic parasite forms (EEFs) was performed using ImageJ software (version 1.53t).

### Liver histopathology

Mouse median liver lobes collected 46 h post sporozoite inoculation were fixed in 4% formaldehyde/PBS (Enzifarma) and paraffin embedded (Leica) in a transversal orientation. Stereology-sectioning into 3 μm thick slices was carried out using a Microtome (RM2145, Leica) and slides were stained with hematoxylin (Bio-optica) and eosin (Merck). Analysis of tissue sections was performed using a NanoZoomer-SQ Digital slide scanner (Hamamatsu Photonics) and included identification of intracellular parasite forms, hepatocellular damage and inflammatory infiltrates.

### Assessment of *P*. *berghei* blood infection

Mouse parasitaemia was quantified daily after injection of *P. berghei*-iRBCs by a bioluminescence assay using a modified version of the protocol described in (Zuzarte-Luis et al., 2014b). Briefly, 5 μL of blood were collected from mice’s tail vein into 45 μL of Firefly luciferase lysis buffer (Biotium) and stored at -20 °C. Luminescence was measured on a Tecan Infinite M200 Plate Reader (Tecan) following the addition of 50 μL of D-luciferin dissolved in Firefly luciferase assay buffer to each well of a 96-well white plate containing 15 μL of the blood lysate, according to the manufacturer’s instructions. Values of luciferase activity are expressed as relative luminescence units (RLU), using blood from non-infected mice as an internal control.

### Blood immunophenotyping

Analysis of circulating immune cell populations was conducted from 10 μL of blood collected from the tail vein into 90 ul of heparin/PBS (10U/mL), at days 2 and 7 post maSCV2 infection. Blood samples were centrifuged at 500 g for 5 min and red blood cells depleted by incubation with an ammonium-chloride-potassium solution (0.15 M NH_4_Cl, 0.01 M KHCO_3_, 0.01 M Na_2_EDTA in double distilled H_2_0) for 4 min at RT. The lysis reaction was stopped by addition of FACS buffer (2% HI-FBS in 1x PBS) and centrifugation, followed by incubation with Fc block (eBiocience) for 10 min at 4-8 °C. The samples were then washed with 1x PBS, centrifuged and surface stained for 20 min at 4-8 °C with the following mix of fluorochrome-conjugated anti-mouse monoclonal antibodies: CD11b Alexa Fluor 488 (clone M1/70), Ly6G PerCP-Cy5.5 (clone 1A8), TCR γδ Brilliant Violet (BV) 421 (clone GL3), CD8α BV 510 (clone 53-6.7), Ly6C BV 605 (clone HK1.4), NK1.1 BV 711 (clone PK136), CD44 BV785 (clone IM7), CD4 APC (clone GK1.5), CD45 Alexa Fluor 700 (clone 30-F11), CD19 PE (clone 1D3), CD62L PE-Dazzle594 (clone MEL-14), PD-1 PE-Cy5 (clone 29F.1A12), CD3 PE-Cy7 (clone 145-2C11), purchased from either Biolegend or Sysmex. A Fixable Viability Dye (eBioscience) was also included for exclusion of dead cells. Samples were subsequently washed with 1x PBS, centrifuged and fixed in 2% formaldehyde/PBS for 15 min at RT, before being retrieved from the BSL-3 facility and further centrifuged and resuspended in FACS buffer. Samples were acquired on a BD LSRFortessa™ X-20 Flow Cytometer and data were analysed using the FlowJo software (version 10.8.1).

### Statistical analyses

Results are represented as mean ± standard deviation (SD) or individual values. Viral titers, parasitaemias and circulating immune cell numbers were transformed into their Log base 10 values for linearization before statistical analyses. Statistical differences in viral titers, liver loads, EEF number and size, and parasitaemias between pairs of experimental groups were assessed by unpaired t tests. Statistical differences in circulating immune cells were analysed by a two-way analysis of variances (ANOVA) followed by Sidak’s test for multiple comparisons between experimental groups, and by paired t tests between days within the same experimental group. Differences in survival rates were analysed by the Mantel-Cox (log rank) test.

## Data availability statement

The original contributions presented in the study are included in the article/**Supplementary Material**. Further inquiries can be directed to the corresponding author.

## Ethics statement

The animal study was approved by Direção Geral de Alimentação e Veterinária (license number: 001878-2021). The study was conducted in accordance with the local legislation and institutional requirements.

## Author contributions

AF: Conceptualization, Formal analysis, Investigation, Methodology, Writing – original draft. AFM: Conceptualization, Formal analysis, Investigation, Methodology, Supervision, Writing – review & editing. DM: Investigation, Methodology, Writing – review & editing. JS: Resources, Writing – review & editing. HN-C: Investigation, Methodology, Writing – review & editing. MP: Conceptualization, Funding acquisition, Project administration, Resources, Supervision, Validation, Writing – review & editing.
