# Supplementary material for: SARS-CoV-2 decreases malaria severity in co-infected rodent models
Source: Front Cell Infect Microbiol. 2023 Dec 13;13:1307553. doi: 10.3389/fcimb.2023.1307553 (PMC10753813; doi:10.3389/fcimb.2023.1307553)
Supplement: Supplementary file 1 [file DataSheet_1.docx]

***Supplementary Material***

**SARS-CoV-2 decreases malaria severity
in co-infected rodent models**

**Ana Fraga^†^, Andreia F. Mósca^†^, Diana Moita, J. Pedro Simas, Helena Nunes-Cabaço, Miguel Prudêncio***

**^†^** These authors share first authorship

*** Correspondence:** Miguel Prudêncio

[mprudencio@medicina.ulisboa.pt](mailto:mprudencio@medicina.ulisboa.pt)

1. **Supplementary Figures**


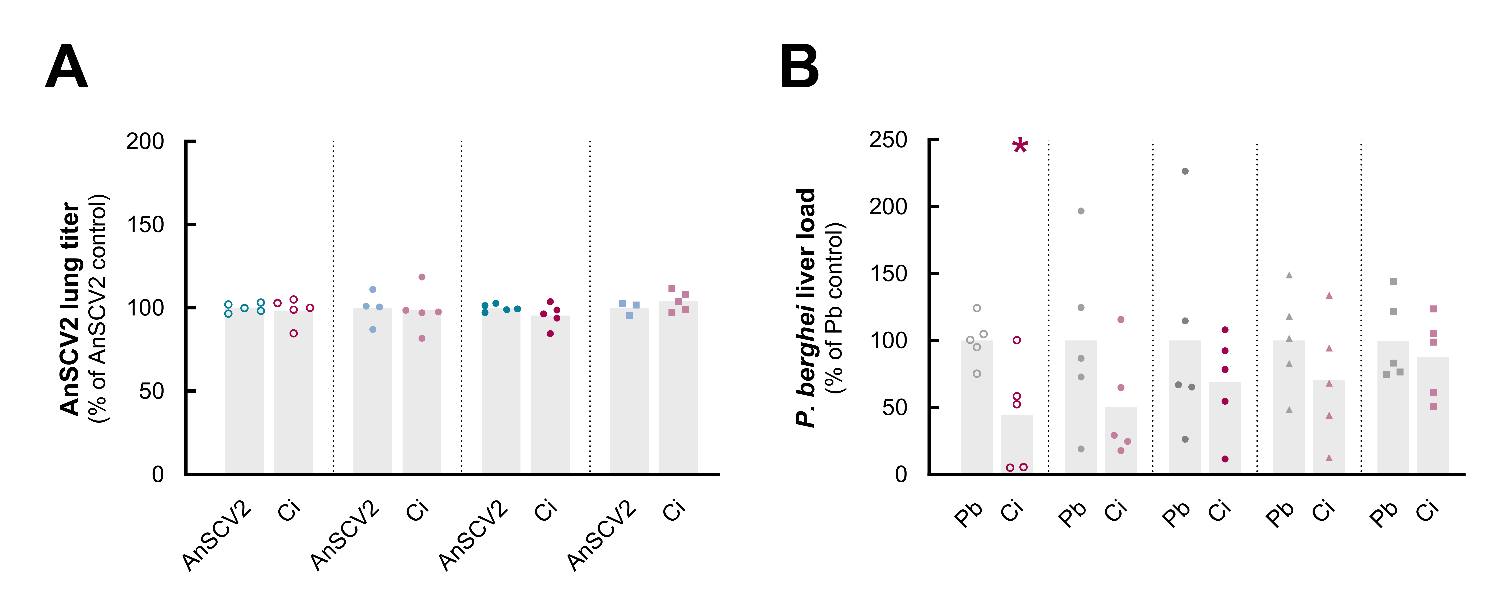


**Figure S1. Viral and parasitic infection quantification.** **(A)** AnSCV2 pulmonary infection quantified by virus titration and plaque-forming assay in Vero CCL-81 cells 4 days after virus inoculation of single-infected mice (AnSCV2 – blue symbols) and mice subsequently infected with 3x10^4^ *P. berghei* sporozoites (Ci – pink symbols). Each symbol represents one mouse and bars represent the mean values for the group in each experiment (n=3-5 mice per group) from four independent experiments. **(B)** *P. berghei* liver infection quantified by RT-qPCR 46 h after sporozoite injection into naïve mice (Pb – grey symbols) or mice exposed to AnSCV2 infection 2 days earlier (Ci – pink symbols). Each symbol represents one mouse and bars represent the mean values for the group in each experiment (n=5 mice per group) from five independent experiments. The statistical significance of differences between groups from the same experimental condition was assessed by employing an unpaired t test (* p<0.05). Coloured asterisks indicate differences relative to the respective single-infected control group.


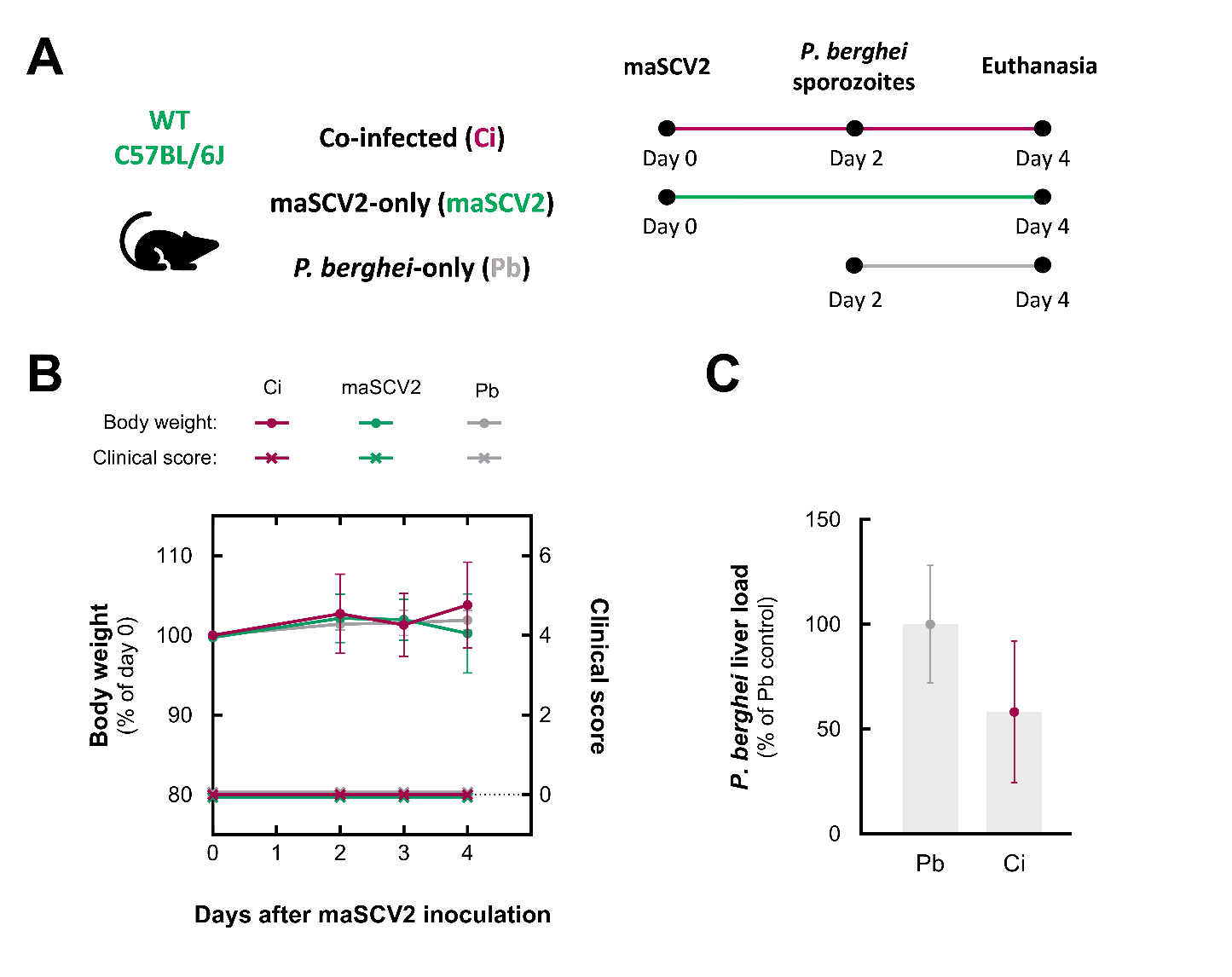


**Figure S2. An ongoing asymptomatic maSCV2 infection does not significantly impact a secondary *P. berghei* liver infection. (A)** Schematic representation of *Plasmodium* liver stage co-infection model. Depicted are the time points of infection with the mouse-adapted SARS-CoV-2 virus strain (maSCV2) and/or *P. berghei* sporozoites, and of euthanasia for organ collection. Experimental groups include mice inoculated with *P. berghei* sporozoites two days after exposure to maSCV2 (Ci – pink), mice solely exposed to maSCV2 infection (AnSCV2 – green), and mice solely exposed to *P. berghei* sporozoite inoculation (Pb – grey). **(B)** Daily monitoring of body weight and signs of disease. Each symbol represents mean values of the group in each time-point and error bars the standard deviation from one experiment (n=5 mice per group). Dotted horizontal lines represent the weight threshold for euthanasia (top graph). **(C)** *P. berghei* liver infection quantified by RT-qPCR 46 h after sporozoite injection. Each symbol represents mean values for the group and error bars the standard deviation from one experiment (n=5 mice per group). maSCV2 pulmonary infection was confirmed by RT-qPCR. The statistical significance of differences between pairs of groups was assessed by a two-way analysis of variance (ANOVA) followed by the Sidak’s test for multiple comparisons in **(B)** and by an unpaired t test in **(C)**.


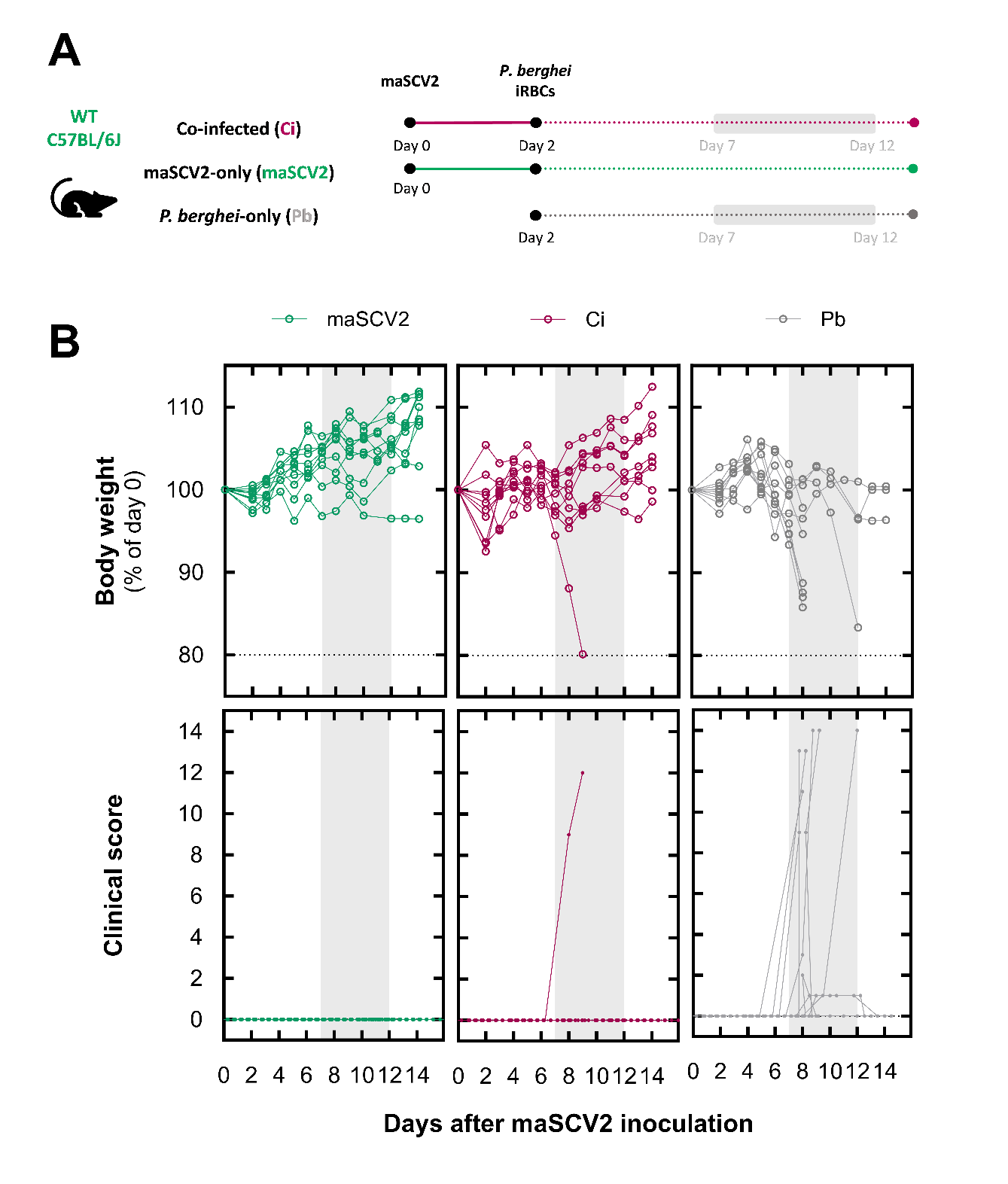


**Figure S3. An attenuated SARS-CoV-2 infection protects against severe malaria pathology.
(A)** Schematic representation of *Plasmodium* blood stage co-infection model. Schematic representation of the attenuated co-infection model. Depicted are the time points of infection with the mouse-adapted SARS-CoV-2 strain (maSCV2) and/or *P. berghei*-infected red blood cells (iRBCs), and the period during which parasitaemia and survival were monitored. Grey-shaded area corresponds to the 5-day window of ECM development. Experimental groups include mice inoculated with *P. berghei*-iRBCs 2 days after maSCV2 exposure (Ci – pink), mice solely exposed to maSCV2 infection (maSCV2 – green), or solely inoculated with *P. berghei*-iRBCs (Pb – grey).
**(B)** Daily monitoring of body weight and signs of disease. Lines represent each individual mouse (n=10 mice per group). Dotted horizontal lines represent the weight threshold for euthanasia (top graph).


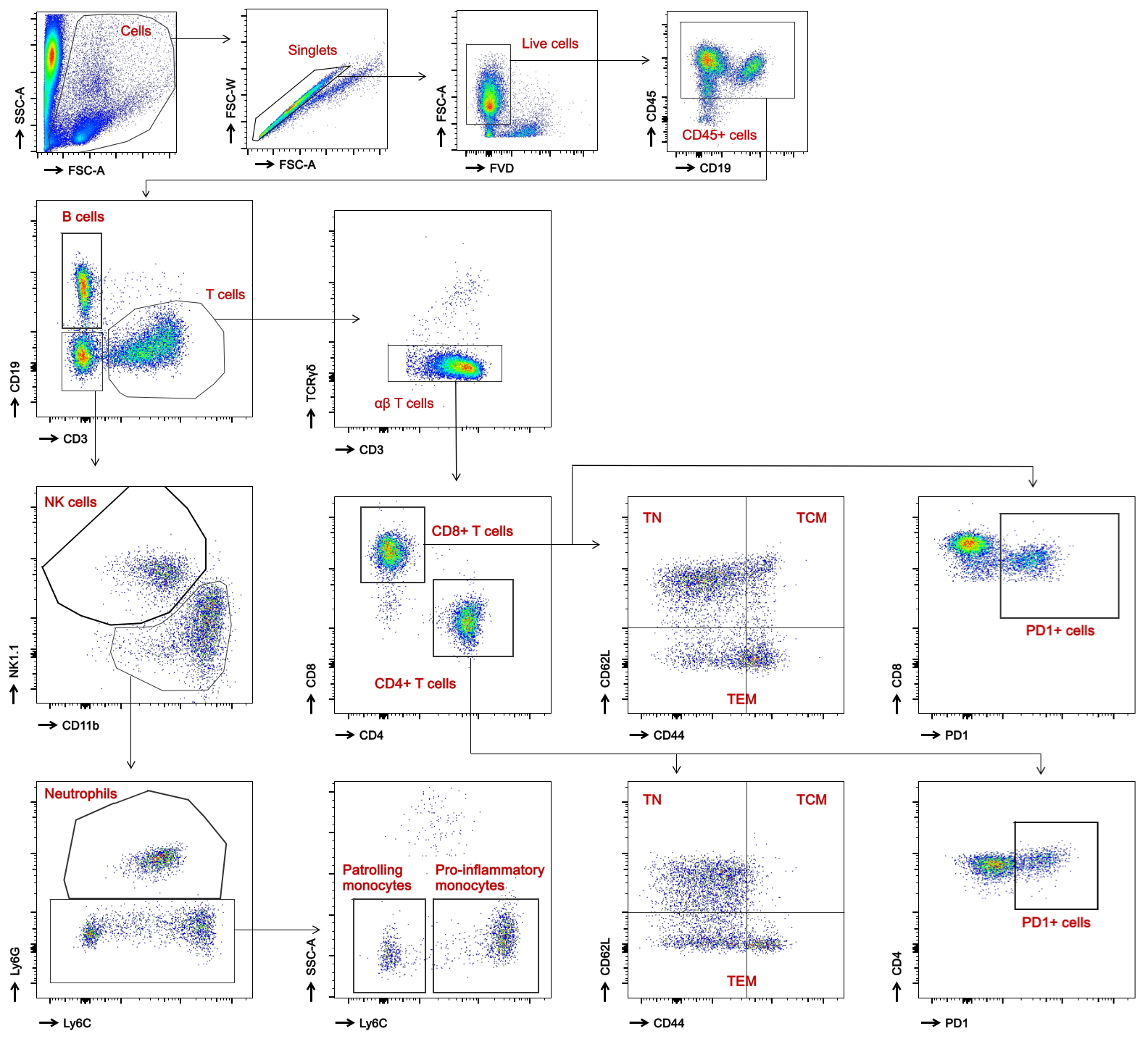


**Figure S4. Representative gating strategy employed in the identification of blood immune populations by flow cytometry.** Single cells were selected based on forward scatter (FSC) and side scatter (SSC) and dead cells were excluded using the fixable viability dye (FVD), followed by leukocyte identification by CD45 marker positivity. Immune cell populations were defined and identified resorting to the following marker staining pattern: B cells (CD3-CD19+),
natural killer cells (NK, CD3negNK1.1+), neutrophils (CD3negCD11b+Ly6G+),
pro-inflammatory monocytes (CD3negCD11b+SSClowLy6Chigh), patrolling monocytes (CD3negCD11b+SSClowLy6Clow), CD4+ T cells (CD3+TCRγδnegCD4+), CD8+ T cells (CD3+TCRγδnegCD8+). Subpopulations within the CD4+ T and CD8+ T cell compartments were further defined as T naïve (T_N_, CD44negCD62L+), central memory (T_CM_, CD44+CD62L+) and effector/effector memory (T_EM_, CD62Lneg), and programmed cell death-1 expressing (PD-1+) cells. SSC-A, side scatter area; FCS-A, forward scatter area; FSC-W, forward scatter width; neg, negative.


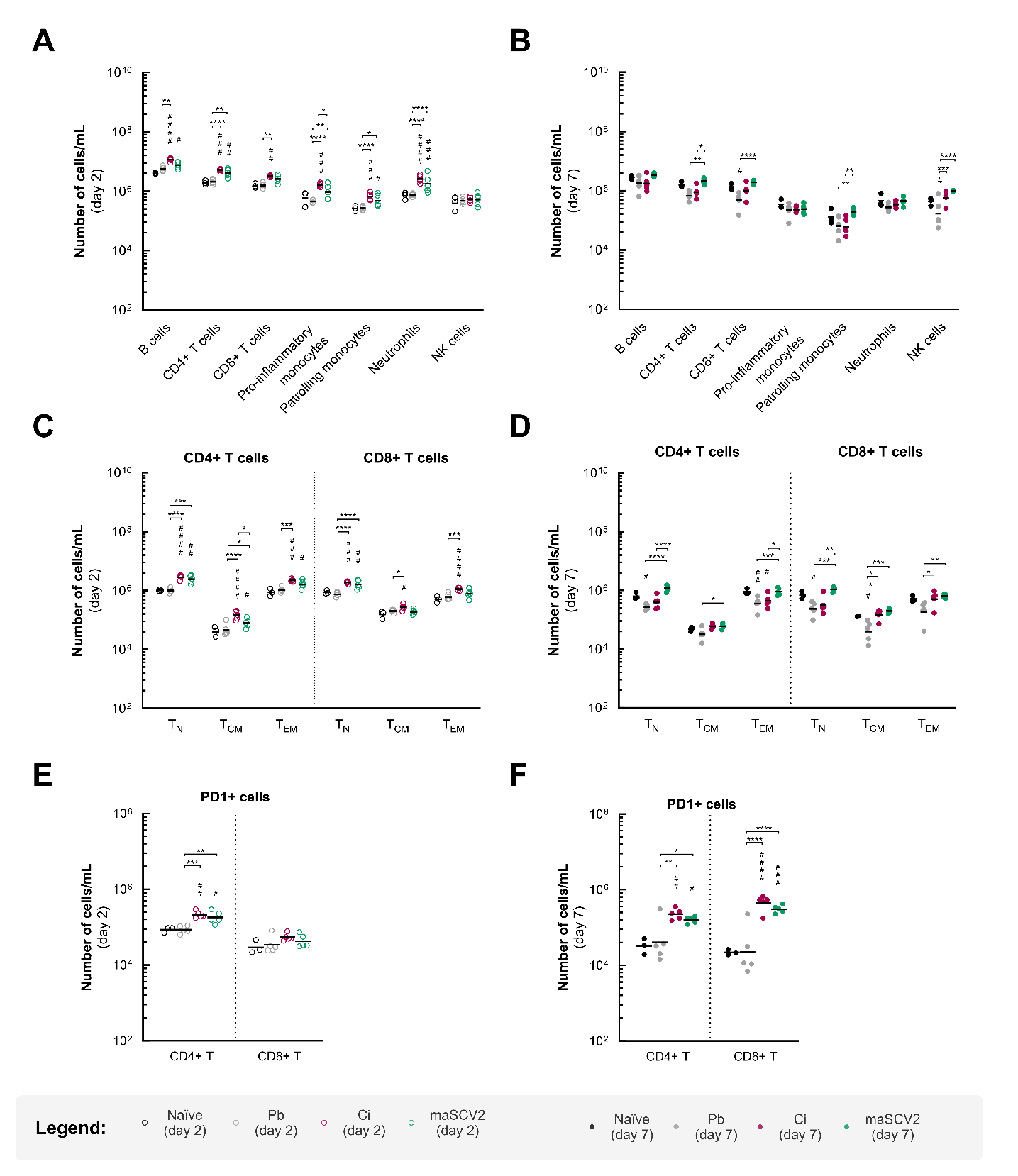


**Figure S5. Impact of maSCV2 and/or *P. berghei* blood infection on the number of circulating immune cells.** Scatter dot plots for the B, CD4+ T, CD8+ T, monocyte, neutrophil and natural killer (NK) cell **(A, B)**, the naïve (T_N_), central memory (T_CM_) and effector/effector memory (T_EM_) CD4+ and CD8+ T cell **(C, D)**, and the PD-1+ CD4+ and CD8+ T cell **(E, F)** population numbers on day 2 **(A, C, E)** and day 7 **(B, D, F)**, in each experimental condition. Experimental groups include naïve mice (black symbols), mice solely infected with *P. berghei*-infected red blood cells (Pb – grey symbols), mice exposed to maSCV2 infection 2 days prior to *P. berghei* inoculation (Ci – pink symbols) and mice only exposed to maSCV2 infection (maSCV2 – green symbols). Each symbol represents one individual mouse and horizontal lines represent the mean value for the group from one experiment (n=4-5 mice per group).The statistical significance of differences relative to the naïve control group (^#^) and between the remaining experimental groups (*) was assessed by a two-way analysis of variance (ANOVA) followed by the Sidak’s test for multiple comparisons (^#/^* p<0.05, ^##/^** p<0.01, ^###/^*** p<0.001, ^####/^**** p<0.0001).


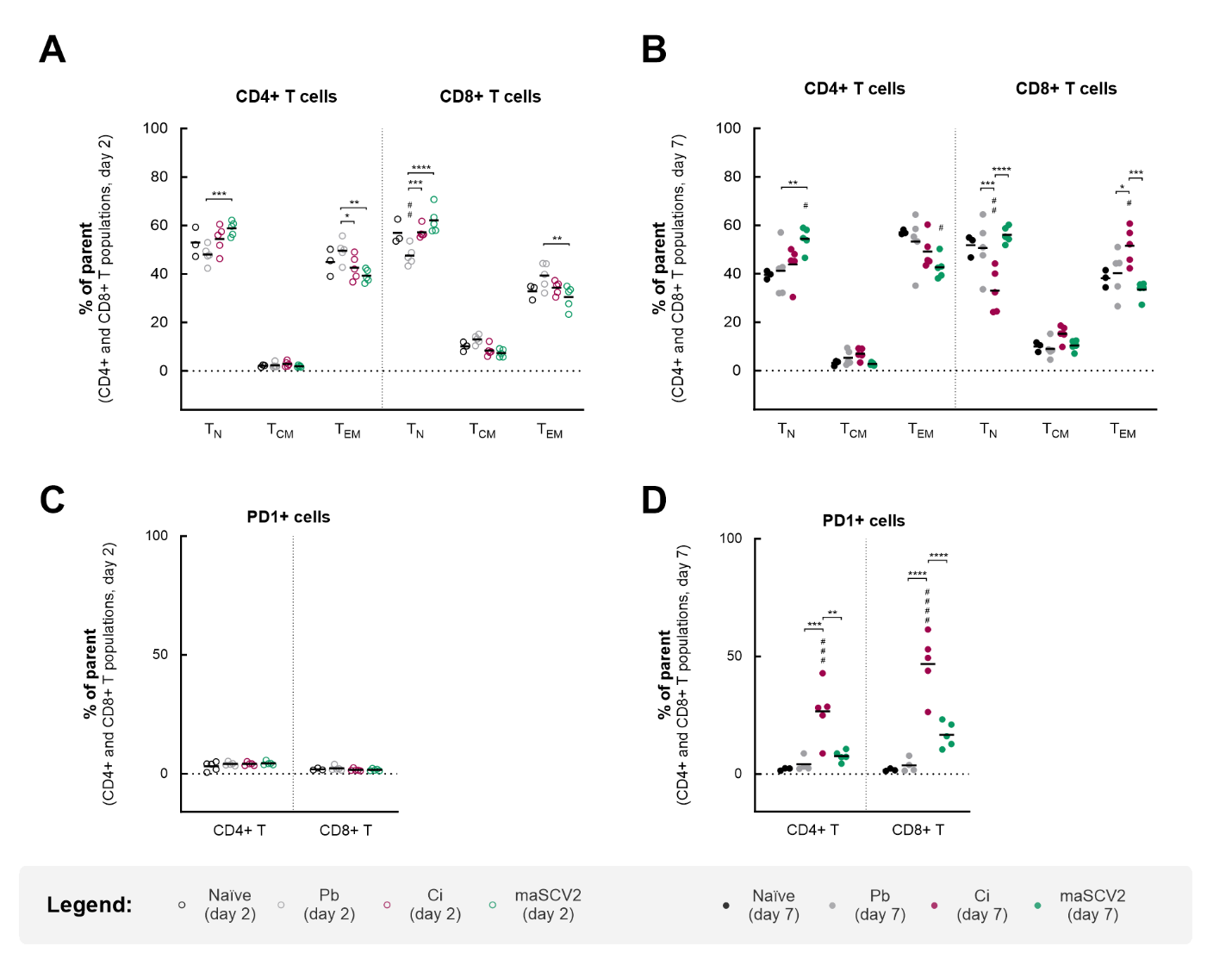


**Figure S6. Impact of maSCV2 and/or *P. berghei* blood infection on the proportion of circulating T cell subsets.** Scatter dot plots for the proportion of naïve (T_N_), central memory (T_CM_) and effector/effector memory (T_EM_) **(A, B)**, and of PD-1+ **(C, D)** cells within the CD4+ and CD8+ T cell compartments on day 2 **(A, C)** and day 7 **(B, D)**, in each experimental condition. Experimental groups include naïve mice (black symbols), mice solely infected with *P. berghei*-infected red blood cells (Pb – grey symbols), mice exposed to maSCV2 infection 2 days prior to *P. berghei* inoculation (Ci – pink symbols) and mice only exposed to maSCV2 infection (maSCV2 – green symbols). Each symbol represents one individual mouse and horizontal lines represent the mean value for the group from one experiment (n=4-5 mice per group). The statistical significance of differences relative to the naïve control group (^#^) and between the remaining experimental groups (*) was assessed by a two-way analysis of variance (ANOVA) followed by the Sidak’s test for multiple comparisons (^#/^* p<0.05, ^##/^** p<0.01, ^###/^*** p<0.001, ^####/^**** p<0.0001).

1. **Supplementary Tables**

**Table S1. Media compositions for Vero CCL-81 cell culture and lung titration.**

| Homogenization medium | Dulbecco's modified Eagle's medium (DMEM, Thermofisher) supplemented with penicillin 50 U/mL and streptomycin 50 µg/mL (Thermofisher) |
| --- | --- |
| Growth  medium | Dulbecco's modified Eagle's medium (DMEM, Thermofisher) supplemented with 10 % heat inactivated-foetal bovine serum (HI-FBS, Thermofisher), penicillin 50 U/mL and streptomycin 50 µg/mL (Thermofisher), and 2 mM glutamine (Thermofisher) |
| Maintenance medium | Dulbecco's modified Eagle's medium medium (DMEM, Thermofisher) supplemented with 2.5 % heat inactivated-foetal bovine serum (HI-FBS, Thermofisher), penicillin 50 U/mL and streptomycin 50 µg/mL (Thermofisher), and 2 mM glutamine (Thermofisher). |
| Overlay  medium | 1.25% carboxymethyl cellulose (Merck) in maintenance medium |

**Table S2. List of primer sequences used for RT-qPCR analyses.**

| Target gene | Forward primer | Reverse primer |
| --- | --- | --- |
| **SARS-CoV-2  N2** | 5’ TTACAAACATTGGCCG CAAA 3’ | 5’ GCGCGACATTCCGA AGAA 3’ |
| ***P. berghei*  18S rRNA** | 5’AAGCATTAAATAAAGCGAA TACATCCTTAC 3’ | 5’ GGAGATTGGTTTTGACGT TTATGTG 3’ |
| **mouse Hprt** | 5’ TTTGCTGACCTGCTGG ATTAC 3’ | 5’ CAAGACATTCTTTCCAGT TAAAGTTG 3’ |
